# Modeling Thixotropic Hydrogel Carriers to Limit Healthy-Tissue Exposure via Localized Drug Retention in Chemotherapy

**DOI:** 10.3390/polym18141704

**Published:** 2026-07-10

**Authors:** Miha Brojan, Jacopo Komic, Enej Istenič

**Affiliations:** Faculty of Mechanical Engineering, University of Ljubljana, 1000 Ljubljana, Slovenia; jacopokomic@gmail.com (J.K.); enej.istenic@fs.uni-lj.si (E.I.)

**Keywords:** coupled multiphysics model, thixotropy, Biot poroelastic medium, Darcy’s law, advection–diffusion, spherical symmetry, hydroxypropyl methylcellulose (HPMC), methotrexate (MTX)

## Abstract

In this work, we develop a coupled multiphysics model that integrates polymer carriers exhibiting time-dependent thixotropic structural recovery with Darcy flow, linear Biot poroelasticity and advection–diffusion transport in a spherically symmetric, isotropic and homogeneous tissue domain. The formulation explicitly links rheological evolution to pressure-driven flow, interstitial deformation and solute transport through a unified framework, enabling systematic prediction of post-injection behavior. Unlike conventional approaches that assume constant carrier properties, the present model incorporates a time-dependent viscosity evolution, capturing the transition from an initially shear-thinned state to a recovered, highly viscous structure. Numerical simulations using hydroxypropyl methylcellulose and methotrexate parameters as representative components demonstrate that rapid post-injection viscosity recovery suppresses pressure-driven transport and diffusion, thereby enhancing local drug retention near the injection site. A systematic sensitivity analysis identifies the equilibrium viscosity as the dominant parameter controlling spatial localization, whereas tissue mechanical properties exert a comparatively minor influence. An effectiveness metric based on the Kullback–Leibler divergence reveals a tumor-size-dependent trade-off between spatial coverage and retention. The proposed framework thus introduces a predictive tool for analyzing coupled rheological-transport interactions and for the rational design and optimization of thixotropy-enhanced local chemotherapy strategies.

## 1. Introduction

Chemotherapy remains one of the most widely used treatments for cancer, but its cytotoxic mechanism is inherently non-selective. Agents designed to kill rapidly proliferating malignant cells also damage healthy tissues with high turnover. The spatial localization of drug delivery is therefore a decisive factor in balancing efficacy against toxicity, motivating systems that confine the active compound to the tumor site.

First applied in the 1940s, chemotherapy acts through several pharmacological classes, most notably alkylating agents, which crosslink and damage DNA, and antimetabolites such as methotrexate, which block nucleotide biosynthesis, ultimately driving proliferating cells toward apoptosis [1,2,3]. Because these mechanisms target cell division rather than tumor identity, fast-dividing healthy tissues (e.g., hair follicles and gastrointestinal epithelium) are also affected, giving rise to characteristic side effects such as alopecia and gastrointestinal toxicity [4,5,6].

These limitations have driven a shift from systemic administration toward localized biomaterial platforms, particularly smart polymeric hydrogels, that shield healthy tissue while providing sustained, site-specific release [7]. Within this class, thixotropic formulations are especially attractive. Their shear-dependent viscosity allows smooth injection yet promotes viscosity recovery at rest, limiting unwanted spread and enhancing retention at the tumor site, while their tunable rheology enables application-specific design. Realizing such systems, however, requires predicting their transient mechanical and transport behavior under physiological conditions. Rather than relying on resource-intensive trial-and-error, in silico design and macroscale numerical modeling have become central to this effort [8]. Developing coupled mathematical frameworks that simultaneously capture carrier physics and interstitial tissue dynamics is thus essential for optimizing localized drug retention and constitutes the focus of the present work.

A range of injectable platforms has been developed to confine chemotherapeutic agents to the tumor site [9]. In situ-gelling systems, most prominently thermoresponsive formulations, are administered as free-flowing solutions that transition to a drug-loaded depot at body temperature [10,11], while composite and depot architectures, such as biodegradable polymeric microspheres and lipid-based carriers enable staged or sustained release of one or more agents [12]. Beyond such passive retention strategies, active tumor-targeting platforms have also been pursued, including platelet-mediated nanocarriers that exploit native tumor recognition to enhance intratumoral accumulation and penetration while remodeling the tumor microenvironment [13]. A recurring motivation across these systems is that a simple intratumoral injection of an unthickened drug solution disperses rapidly, lowering the local dose and exposing healthy tissue. Thixotropic formulations address this through a purely physical mechanism: shear-thinning permits facile injection, while rest-state structural recovery restores viscosity and limits the spread. The behavior of such systems is, however, almost exclusively characterized experimentally and existing transport models of local chemotherapy generally assume constant carrier properties. The present work differs in explicitly incorporating time-dependent thixotropic structural recovery within a coupled poroelastic transport framework, enabling systematic prediction of how post-injection viscosity evolution shapes drug retention and spatial distribution.

Thixotropy is most commonly observed in dispersions and represents a macroscopic rheological response arising from complex, shear-induced changes in the material’s microstructure. Although a universal constitutive model applicable to all thixotropic fluids remains under development, the underlying mechanisms are generally well understood. At rest, relatively weak intermolecular interactions promote the formation of transient, organized structures within the fluid. Upon the application of shear, these structures are disrupted by hydrodynamic forces, leading to a reduction in viscosity, while their gradual reformation occurs once the shear is removed. The primary interactions governing this behavior include van der Waals forces, electrostatic (Coulombic) interactions, hydrogen bonding, steric effects and Brownian motion. Mewis and Wagner [14] provide one of the most comprehensive treatments of thixotropy, covering its physical origins, definitions and modeling strategies; their work has strongly informed the present study. Larson [15] develops constitutive equations for thixotropic fluids; his simplified formulation for ideal systems is adopted here as a practical starting point due to its analytical tractability. In addition, Schotting, Moser and Hassanizadeh [16] extend the advection–diffusion framework to account for high concentration gradients. The formulation proposed in this work therefore combines concepts from injectable hydrogel systems and fluid transport in biological tissues to describe the behavior of thixotropic drug carriers. Notably, the case of Newtonian fluids with constant viscosity can be regarded as a limiting approximation within this framework.

This paper presents a spherically symmetric model describing the spread of a chemotherapeutic agent from the injection site, in which thixotropic properties are incorporated into the formulation. The derivation is theoretical and intentionally generic, allowing application to a wide range of fluids and surrounding media; hydroxypropyl methylcellulose (HPMC) is investigated as a primary case study to demonstrate the model’s utility. The model is based on the advection–diffusion equation, yielding a time- and space-dependent concentration profile of the active substance. The advective component is derived from constitutive equations for ideal thixotropic fluids, while tissue poroelasticity is accounted for through Biot’s equations and Darcy’s law; diffusion is described using the Stokes–Einstein–Sutherland relation. Representative parameter values are applied to illustrate model behavior and the relative contributions of advection and diffusion are subsequently analyzed. To the authors’ knowledge, existing models of local chemotherapy transport generally assume constant carrier properties, whereas the present work explicitly incorporates time-dependent thixotropic structural recovery within a coupled poroelastic transport framework. This approach enables a systematic evaluation of how post-injection viscosity evolution affects drug retention and spatial distribution.

The remainder of the paper is organized as follows. Section 2 outlines the governing equations and overall model formulation. Section 3 presents the simulation results and their discussion. Finally, conclusions are drawn in Section 4.

## 2. Methods

### 2.1. Theoretical Background

#### 2.1.1. Thixotropy

A commonly used constitutive model for thixotropic fluids expresses the stress tensor σ as proportional to the strain rate tensor $\dot{\varepsilon }$ through a scalar function *f* [15]:

(1) $$\sigma =2f\dot{\varepsilon }.$$

The function *f* depends on the structural parameter Λ and the second invariant of $\dot{\varepsilon }$, which reduces to the shear rate $\dot{\gamma }$ in simple shear flow. It is defined as

(2) $$f=\frac{\sigma_{y}}{\dot{\gamma }}+\mu ,$$

where $\sigma_{y}$ is the yield stress and μ is the dynamic viscosity of the fluid [15].

The evolution of the structure parameter Λ∈[0,1] is governed by

(3) $$\frac{d\Lambda }{dt}=h_{+}\left(1-\Lambda \right)-h_{-}\dot{\gamma }\Lambda ,$$

where $h_{+}$ and $h_{-}$ represent the rates of structure build-up and breakdown, respectively. The viscosity is then related to the structural state through

(4) $$\mu =\mu_{0}\Lambda^{n},$$

where $\mu_{0}$ is the reference viscosity and n∈[0,1] characterizes the sensitivity of viscosity to structural changes [14].

#### 2.1.2. Diffusivity

The diffusion coefficient *D* is derived from the Stokes–Einstein–Sutherland relation:

(5) $$D_{s}=\theta k_{B}T,$$

where θ denotes particle mobility, $k_{B}$ is the Boltzmann constant and *T* is the temperature. For spherical particles in the low-Reynolds-number limit (creeping flow), the mobility is inversely related to the drag coefficient ζ:

(6) $$\theta =\frac{1}{\zeta },$$

with the drag coefficient given by Stokes’ law,

(7) ζ=6πRμ,

where *R* is the particle radius and μ is the dynamic viscosity of the fluid. Substitution yields the diffusion coefficient in the fluid phase,

(8) $$D_{f}=\frac{k_{B}T}{6\pi R\mu }.$$

To account for diffusion within porous biological tissue, this expression is modified by structural factors, including tortuosity τ and constrictivity δ. Since transport occurs within the fluid-filled fraction of the tissue, the porosity dependence can be reformulated accordingly, resulting in the effective diffusion coefficient

(9) $$D=\frac{\delta }{\tau }\frac{k_{B}T}{6\pi R\mu }.$$

#### 2.1.3. Darcy’s Law

Fluid flow through biological tissue can be described using Darcy’s law, which relates the superficial velocity q to the pressure gradient ∇p, accounting for the tissue permeability *k* and fluid viscosity μ:

(10) $$q=-\frac{k}{\mu }\nabla p.$$

#### 2.1.4. Biot Poroelasticity

For a linear, isotropic poroelastic medium undergoing small deformations, the quasi-static equilibrium of the solid–fluid mixture is described by Biot’s equations:

(11) ∇·σ=0,

(12) $$\sigma =\sigma^{\prime }-\alpha pI,$$

where $\sigma^{\prime }$ denotes the effective stress in the solid skeleton, *p* is the pore pressure and α is the Biot–Willis coefficient. For a linear elastic, drained skeleton, the effective stress is given by

(13) $$\sigma^{\prime }=\lambda \varepsilon_{v}I+2G\varepsilon ,$$

where λ is the drained Lamé parameter, *G* is the shear modulus and $\varepsilon_{v}$ is the volumetric strain. Mass conservation of the pore fluid, combined with Darcy’s law, yields

(14) $$\frac{1}{M_{B}}\frac{\partial p}{\partial t}+\alpha \frac{\partial \varepsilon_{v}}{\partial t}=\nabla \cdot \left(\frac{k}{\mu }\nabla p\right),$$

where $M_{B}$ is the Biot modulus (inverse storage coefficient) [17].

#### 2.1.5. Advection–Diffusion Equation

Since the active substance in the chemotherapy formulation is transported by both advection and diffusion, its concentration *C* is modeled using the advection-diffusion equation:

(15) $$\frac{\partial C}{\partial t}=\nabla \cdot \left(D\nabla C-vC\right)+S,$$

where v denotes the advective fluid velocity and *S* represents source or sink terms.

### 2.2. Selection and Characterization of the Thixotropy-Inducing Polymer

The thixotropic response assumed in Section 2.1.1 must be supplied by a specific additive. In this paper, we assign this role to hydroxypropyl methylcellulose (HPMC), a non-ionic, semi-synthetic cellulose ether obtained by partial substitution of the cellulose hydroxyl groups with methoxy and hydroxypropoxy groups [18,19]. HPMC was selected for three reasons. First, it is biocompatible, biodegradable, water-soluble and stable across a broad physiological pH range, and classified as *Generally Recognized As Safe* (GRAS), with established pharmacopoeial use as a viscosity modifier and matrix former in injectable and controlled-release formulations. Second, it is inexpensive and available in pharmaceutical grades spanning several orders of magnitude in solution viscosity, so that the rheological parameters of the formulation can be tuned through the choice of polymer grade and concentration. Third, its thixotropic, shear-thinning response arises from a physically reversible, hydrogen-bonded entanglement network, precisely the behavior described by the structural-parameter model adopted in Equations (1)–(4). The specific grade considered here is Methocel Type K, a commercial HPMC of the 2208 substitution type (approximately 19–24% methoxy and 4–12% hydroxypropoxy groups), which hydrates readily in aqueous media to form a highly viscous, entangled network whose strength scales with polymer concentration and molecular weight. The K-series spans a wide range of viscosity grades (e.g., K4M, K15M, K100M) that are widely employed in controlled-release dosage forms, since higher-viscosity grades form stronger, stable hydrophilic matrices with greater resistance to mass transport, thereby reducing drug diffusion and prolonging retention at the delivery site. Together with its mucoadhesive, non-toxic and non-ionic character, this has established Methocel K as one of the most widely used polymers for sustained and localized drug delivery [19]. Accordingly, it is treated in the present study as a thixotropic carrier matrix whose viscosity and structural-recovery characteristics govern the post-injection distribution and retention of the therapeutic agent within tumor tissue.

A further practical requirement is injectability, which is inherently coupled. The formulation must flow through the needle under high shear yet recover sufficient viscosity within the tissue to limit spread. In the present model, this is represented implicitly, with the formulation entering the tissue in a shear-broken, low-viscosity state $\mu_{N}$ and subsequently recovering toward its equilibrium value $\mu_{0}$. A detailed treatment of injectability, such as that presented in [20], lies outside the scope of this study and is identified as an important subject for future work.

At the molecular level, the macroscopic thixotropy of an HPMC solution originates from the mechanisms summarized in Section 1. Under shear, hydrodynamic forces disrupt the hydrogen-bonded, entangled cellulose-ether network and the apparent viscosity falls; once shear is removed, thermal motion and inter-chain hydrogen bonding re-establish the network and the viscosity recovers. The degree of methoxy and hydroxypropoxy substitution and the polymer molecular weight govern the strength of inter-chain association and hence the equilibrium viscosity, while the polymer concentration relative to the entanglement threshold governs both the magnitude and the rate of the structural response [19]. This molecular picture maps directly onto the three rheological parameters of the model. The fully recovered equilibrium viscosity $\mu_{0}$ in Equation (4) corresponds to the apparent viscosity of the structured HPMC solution at rest and is set primarily by polymer grade and concentration; in this work we adopt $\mu_{0}=4.0$ Pa·s as a representative value for a readily injectable HPMC formulation [19], while the sensitivity analysis in Section 3.3 explores the full clinically accessible range up to 100 Pa·s. The exponent *n* in Equation (4), taken as n=0.7, parametrizes the strength of the coupling between the viscosity and the structural parameter Λ; it should not be confused with the Ostwald–de Waele flow-behavior index commonly reported for the steady shear-thinning of HPMC, which is a distinct quantity describing the stress-shear-rate relationship rather than the structure-viscosity coupling. The structure build-up coefficient $h_{+}$ in Equation (3), taken as $h_{+}=0.10$ s^−1^, corresponds to a characteristic structural-recovery time $1/h_{+}=10$ s. This short timescale reflects the rapid network reformation typical of cellulose-ether solutions and is consistent with the near-complete viscosity recovery observed within the first minute after injection and presented in Section 3.1. The injection process itself justifies the assumption, used in Section 2.3, that the formulation enters the tissue in an essentially unstructured, low-viscosity state. During passage through the needle, the polymer network experiences shear rates several orders of magnitude higher than those subsequently generated by the slow pressure-driven flow in the tissue; the network is therefore fully broken down at the moment of delivery and the injection-state viscosity is taken equal to that of the underlying Newtonian solvent $\mu_{N}$. After deposition, the negligible interstitial shear allows the structure to rebuild, driving the viscosity from $\mu_{N}$ back toward $\mu_{0}$.

Two further simplifications specific to HPMC are adopted. First, below its thermal gelation temperature, an aqueous HPMC solution is shear-thinning but does not exhibit a measurable yield stress; accordingly, the yield-stress term $\sigma_{y}$ in Equation (2) is set to zero and the formulation is treated as a purely thixotropic, shear-thinning fluid rather than a yield-stress material. Second, the active substance is treated as a passive solute, so that any specific hydrogen-bonding interaction between methotrexate and the HPMC matrix is neglected, which is consistent with experimental observations [21]. Because HPMC matrices are known to retard the release and diffusion of incorporated drugs [19], this assumption is conservative: accounting for drug-polymer interaction would further reduce the predicted spread, reinforcing rather than weakening the localization effect that is the central result of this study.

### 2.3. Mathematical Model of Drug Delivery

As established, the distinction between fluids within the proposed framework arises primarily from their viscosity, whether constant or time-dependent. Accordingly, the model formulation is centered on this property.

The analysis is initiated at the moment the final fluid element exits the syringe tip, at which point the injected formulation occupies a finite volume within the tissue. This initial volume, $V_{0}$, is related to the injected volume *V* and the tissue porosity φ:

(16) $$V_{0}=\frac{V}{\varphi }.$$

Assuming a spherically symmetric distribution, the corresponding initial bolus radius $r_{0}$ is given by

(17) $$r_{0}={\left(\frac{3V}{4\pi \varphi }\right)}^{1/3}.$$

Once the fluid enters the tissue, shear forces become negligible compared to those experienced within the syringe during injection. It is therefore reasonable to assume that structure breakdown due to shear, represented by the shear rate $\dot{\gamma }$, can be neglected. Under this assumption, the evolution equation for the structure parameter Λ simplifies to

(18) $$\frac{d\Lambda (t)}{dt}=h_{+}\left(1-\Lambda \right).$$

Equation (18) has the solution

(19) $$\Lambda \left(t\right)=1-\Omega e^{-h_{+}\;t},$$

where Ω is the integration constant. Assuming that the thixotropic and Newtonian fluid have the same initial viscosity $\mu_{N}$ (which is a reasonable approximation given the strong shear conditions within the syringe that temporarily suppress structural effects), we can apply

(20) $$\Lambda \left(t=0\right)=\Lambda_{0},$$

(21) $$\mu \left(\Lambda =\Lambda_{0}\right)=\mu_{N}$$

and finally express Λ as

(22) $$\Lambda \left(t\right)=1-\left(1-\sqrt[{n}]{\frac{\mu_{N}}{\mu_{0}}}\right)e^{-h_{+}\;t}.$$

From here and Equation (4), the time dependent viscosity of the thixotropic fluid can be obtained:

(23) $$\mu \left(t\right)=\mu_{0}{\left(1-\left(1-\sqrt[{n}]{\frac{\mu_{N}}{\mu_{0}}}\right)e^{-h_{+}t}\right)}^{n}.$$

The particle radius $R_{p}$ of the active substance is estimated assuming spherical, homogeneous particles. It can be derived from the molar mass *M*, density ρ and Avogadro’s number $N_{A}$ as

(24) $$V_{p}=\frac{m}{\rho }=\frac{M}{N_{A}\rho },$$

which yields

(25) $$R_{p}={\left(\frac{3M}{4\pi N_{A}\rho }\right)}^{1/3}.$$

Substituting this expression into the Stokes–Einstein–Sutherland relation leads to the time-dependent diffusion coefficient

(26) $$D\left(t\right)=\frac{\delta }{\tau }\frac{k_{B}T}{3}{\left(\frac{1}{6\pi^{2}}\frac{N_{A}\rho }{M}\right)}^{1/3}\frac{1}{\mu (t)},$$

highlighting the inverse dependence of diffusion on the evolving viscosity μ(t).

At this stage, the Biot equations are adapted to the spherically symmetric configuration of the problem. Combining Equations (11)–(13) yields the radial relation

(27) $$\frac{\partial \varepsilon_{v}\left(r,t\right)}{\partial r}=\frac{\alpha }{\lambda +2G}\frac{\partial p(r,t)}{\partial r}.$$

Integrating with respect to *r* and differentiating with respect to *t*, the resulting expression can be substituted into the spherically symmetric form of Equation (14), giving

(28) $$\frac{1}{M_{B}}\frac{\partial p(r,t)}{\partial t}+\frac{\alpha^{2}}{\lambda +2G}\frac{\partial p(r,t)}{\partial t}=\frac{1}{r^{2}}\frac{\partial }{\partial r}\left(r^{2}\frac{k}{\mu (t)}\frac{\partial p(r,t)}{\partial r}\right).$$

The coefficients on the left-hand side can be combined into an effective storage parameter *c*, leading to

(29) $$c=\frac{1}{M_{B}}+\frac{\alpha^{2}}{\lambda +2G}\approx \frac{1}{\lambda +2G}$$

and yielding the governing equation for the pressure evolution p(r,t),

(30) $$c\;\frac{\partial p(r,t)}{\partial t}=\frac{1}{r^{2}}\frac{\partial }{\partial r}\left(r^{2}\frac{k}{\mu (t)}\frac{\partial p(r,t)}{\partial r}\right).$$

In Equation (29), incompressibility of both fluid and solid constituents is assumed, allowing the Biot modulus $M_{B}$ and the Biot–Willis coefficient α to be neglected [22]. Under this assumption, the expression simplifies to the form given above.

In spherical coordinates, Darcy’s law provides the superficial velocity from the pressure field p(r,t). Since the advective fluid velocity is related to the superficial velocity through the tissue porosity φ, it follows that

(31) $$v\left(r,t\right)=-\frac{k}{\varphi }\frac{1}{\mu (t)}\frac{\partial p(r,t)}{\partial r}.$$

Finally, the advection–diffusion equation is expressed in spherical symmetry to describe the spatial-temporal evolution of the drug concentration C(r,t):

(32) $$\frac{\partial C(r,t)}{\partial t}=\frac{1}{r^{2}}\frac{\partial }{\partial r}\left(r^{2}\left(D\left(t\right)\frac{\partial C(r,t)}{\partial r}-v\left(r,t\right)C\left(r,t\right)\right)\right)+S_{0}C\left(r,t\right).$$

Here, $S_{0}$ represents the first-order decay constant, related to the biological half-life $t_{1/2}$ by

(33) $$S_{0}=-\frac{\ln2}{t_{1/2}}.$$

The proposed model is therefore governed by Equations (23), (26), and (29)–(33). Notably, the formulation is applicable to both Newtonian and thixotropic fluids, with differences arising solely through the viscosity term μ(t).

### 2.4. Boundary and Initial Conditions of the Drug Delivery Model

Now that all the parameters are known, we need to define our problem’s boundary and initial conditions. Both the Biot and the advection–diffusion equations contain a second derivative of the spatial variable and a first derivative of the temporal variable, therefore two boundary conditions and one initial condition are required for each. The first boundary condition states that at the point of injection, there is no flow of the observed quantity, otherwise the symmetry would be broken:

(34) $$\frac{\partial p}{\partial r}\left(r=0,t\right)=0,$$

(35) $$\frac{\partial C}{\partial r}\left(r=0,t\right)=0.$$

The second boundary condition specifies that, asymptotically, the value of the transported quantity vanishes. In the limit as r→∞, the concentration (and analogously the pressure perturbation) approaches zero, i.e., the system relaxes to its undisturbed state

(36) p(r=∞,t)=0,

(37) C(r=∞,t)=0.

The initial conditions are defined such that the observed quantities assume a finite value within the injected region and vanish outside it. Specifically, both concentration and pressure are prescribed as uniform for $r\leq r_{0}$ and zero for $r>r_{0}$. While this definition is straightforward for concentration, the corresponding pressure field requires additional derivation. Integrating Equation (27) with respect to *r* and combining it with Equation (29), under the assumption α≈1, yields the relation

(38) $$\varepsilon_{v}\left(r,t\right)=cp\left(r,t\right),$$

which links volumetric strain to pore pressure.

We know that at t=0 the volumetric strain is

(39) $$\varepsilon_{v}=\frac{V}{V_{0}}=\varphi ,$$

where Equation (16) has been used to relate the occupied volume to the injected quantity. It should be noted that the injected bolus not only occupies a region of volume $V_{0}$ but also induces dilation of the surrounding fluid-saturated tissue by the injected volume *V*, resulting in a volumetric strain $\varepsilon_{v}=\varphi$. An important consequence of this formulation is that the peak pressure is independent of the injected volume *V*. While *V* determines the initial bolus radius $r_{0}$, the corresponding pressure increase depends solely on the mechanical properties of the tissue. The initial conditions can thus be defined as follows:

(40) $$p\left(r,t=0\right)=\left\{\begin{array}{cc} \frac{\varphi }{c}, & r\leq r_{0},\\ 0, & r>r_{0}, \end{array}\right.$$

(41) $$C\left(r,\;t=0\right)=\left\{\begin{array}{cc} C_{0}, & r\leq r_{0},\\ 0, & r>r_{0}. \end{array}\right.$$

### 2.5. Numerical Implementation

The governing equations derived in Section 2.3 were solved numerically using a custom program built in C. Owing to the assumption of spherical symmetry, the problem reduces to a one-dimensional radial domain extending from the injection point to a sufficiently large outer radius approximating far-field conditions. The spatial domain was discretized using a uniform grid and temporal integration was performed with a constant timestep. Both the pressure equation arising from the Biot–Darcy formulation and the advection–diffusion equation governing drug transport were discretized implicitly to ensure numerical stability over the extended simulation times considered.

At each timestep, the viscosity of the thixotropic formulation was updated according to the rheological model described in Section 2.3. The corresponding diffusion coefficient was then recalculated using the Stokes–Einstein–Sutherland relation. The pressure field was obtained by solving the discretized poroelastic equation, from which interstitial velocities were evaluated and subsequently used in the transport computation.

Drug transport was solved using a conservative finite-volume formulation in spherical coordinates. Diffusive fluxes were evaluated at cell interfaces, while advective transport was discretized using a first-order upwind scheme to ensure numerical stability and suppress non-physical oscillations. Substance decay was incorporated directly into the transport equation via the source term. Discretization of both the pressure and transport equations yielded tridiagonal systems of linear algebraic equations, which were solved efficiently at each timestep using the Thomas algorithm.

The initial pressure and concentration fields were prescribed according to the conditions defined in Section 2.4. Symmetry conditions were imposed at the injection point, while zero pressure and zero concentration were enforced at the outer boundary. Numerical consistency was assessed by monitoring the total mass of the active substance within the computational domain throughout the simulation; the relative mass conservation error is reported in Appendix A.

Simulations were performed with a uniform spatial step of $10^{-4}\;m$. A timestep of $10^{-3}\;s$ was used for short-time analysis (first minute), whereas a timestep of 1 s was adopted for longer simulations. The spatial domain extended to 50 mm for the short-time analysis and 200 mm for longer-time simulations, ensuring sufficient domain size for pressure evolution. To prevent numerical instabilities, the initial conditions were smoothed using a hyperbolic tangent function. Grid convergence was assessed via Richardson extrapolation, with the results presented in Appendix B.

## 3. Results and Discussion

With the model formulation complete, its performance can be evaluated using representative parameter values corresponding to realistic material properties and operating conditions. The short-time dynamics are first examined over the initial minute following injection, a period during which the thixotropic structure is expected to recover toward equilibrium. Subsequently, the evolution of pressure and concentration fields is analyzed over longer timescales.

The parameter values used in the simulations are summarized in Table 1. In both cases, methotrexate (MTX) is taken as the active compound [23], while thixotropy is introduced through the addition of hydroxypropyl methylcellulose (HPMC) [18,19]. In clinical practice, methotrexate is administered as an aqueous solution rather than in crystalline form, typically prepared at concentrations of approximately 25 mg/mL and diluted into standard intravenous carriers such as 0.9 % sodium chloride or 5 % dextrose [24]. In the present model, however, the density ρ refers to the intrinsic (crystalline) density of methotrexate, which is used solely to estimate the molecular volume and corresponding hydrodynamic radius in Equation (25). It should therefore be distinguished from the effective density of the dilute injected solution. Methotrexate was adopted as the representative agent because it is a well-characterized, clinically established antimetabolite for which the physicochemical and pharmacokinetic parameters required by the model (molar mass, density and biological half-life) are reliably documented, enabling a transparent parameterization. Because the active substance is treated as a passive solute, the framework is not specific to methotrexate. Provided drug-polymer interactions do not substantially alter transport, the same formulation applies to other agents through their respective molecular volume, diffusivity and clearance kinetics.

The values reported in Table 1 are representative of those found in the literature, while the simulations employ the exact tabulated values.

For interpretive clarity, the principal parameters carry direct biological meaning: the porosity φ represents the fluid-accessible fraction of the interstitium available for transport and the permeability *k* quantifies the hydraulic conductivity of this interstitial space while the tortuosity τ and constrictivity δ encode the geometric hindrance imposed by the porous tissue matrix on diffusing solutes. The drained Lamé parameter λ and shear modulus *G* characterize the mechanical stiffness of the solid skeleton, while the biological half-life $t_{1/2}$ summarizes in-vivo clearance through metabolism and perfusion. It should be noted that the structural exponent *n* and build-up rate $h_{+}$ are not fully constrained by available data. Their values are adopted as physically reasonable estimates and their influence is presented more thoroughly in Section 3.3.

### 3.1. First Minute

**Figure 1:**
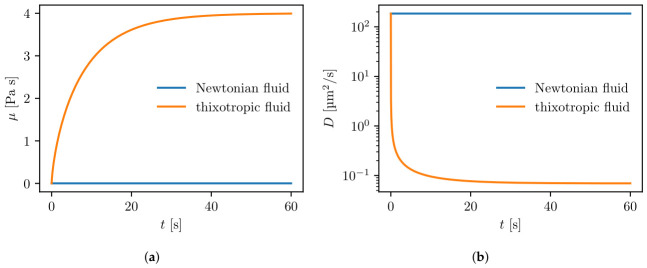
(**a**) Time dependence of the viscosities of the Newtonian and thixotropic medication in the first minute after the injection. (**b**) Time dependence of the fluids’ diffusivities of the Newtonian and thixotropic medication in the first minute after the injection.

The injection process initially distributes the formulation within a spherical region of radius $r_{0}=11.68$ mm. Beyond this point, the transport behavior is primarily governed by the rheological properties of the fluid. The Newtonian formulation maintains a constant viscosity of $\mu_{N}=1.5\times 10^{-3}$ Pa·s, whereas the thixotropic formulation undergoes rapid structural recovery, leading to a significant increase in viscosity from its injection-state value toward the equilibrium value of $\mu_{0}=4.0$ Pa·s. As shown in Figure 1a, the viscosity reaches $\mu_{T}=3.99$ Pa·s after one minute, indicating that structural rebuilding is essentially complete on this timescale.

The increase in viscosity has a direct impact on molecular diffusion. As indicated by Equation (26), diffusivity is inversely proportional to viscosity. Consequently, the progressive structural recovery of the thixotropic formulation leads to a pronounced reduction in the diffusion coefficient. As shown in Figure 1b, the diffusivity of the Newtonian formulation remains constant at $D_{N}=185.05$ µm^2^/s, whereas the diffusivity of the thixotropic formulation decreases to $D_{T}=6.95\times 10^{-2}$ µm^2^/s after one minute. This corresponds to a reduction of more than three orders of magnitude, highlighting the strong influence of thixotropy on drug mobility.

**Figure 2:**
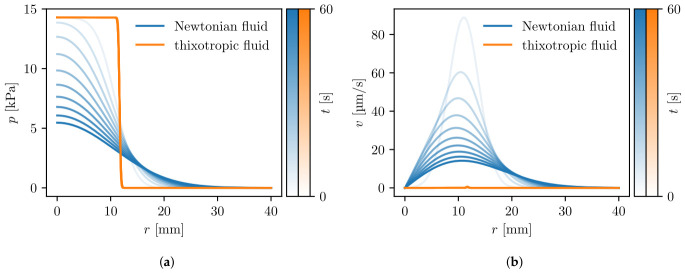
(**a**) Pressure evolution in the Newtonian and thixotropic medication on the radius from the injection point in the first minute after the injection; the first line corresponds to 6 s after injection, the second to 12 s, etc. (**b**) Advection velocity fields of the Newtonian and thixotropic medication on the radius from the injection point in the first minute after the injection; the first line corresponds to 6 s after injection, the second to 12 s, etc.

The pressure evolution shown in Figure 2a exhibits a similar trend. In the Newtonian case, the relatively low viscosity allows pressure disturbances induced by injection to propagate rapidly through the porous medium, resulting in broader pressure distributions. In contrast, the elevated viscosity of the thixotropic formulation restricts pressure propagation, producing a steeper spatial gradient localized near the injection site. Although pressure dissipates over time in both cases, the dissipation is markedly slower for the thixotropic formulation due to its reduced mobility.

The resulting advection velocity fields are shown in Figure 2b. Initially, pressure gradients drive outward flow from the injection site; however, the magnitude of this flow is strongly influenced by viscosity. The Newtonian formulation exhibits significantly higher interstitial velocities across the domain, leading to more pronounced advective transport. In contrast, the thixotropic formulation rapidly attenuates fluid motion, reducing the velocity field and thereby limiting the outward transport of the active substance.

**Figure 3:**
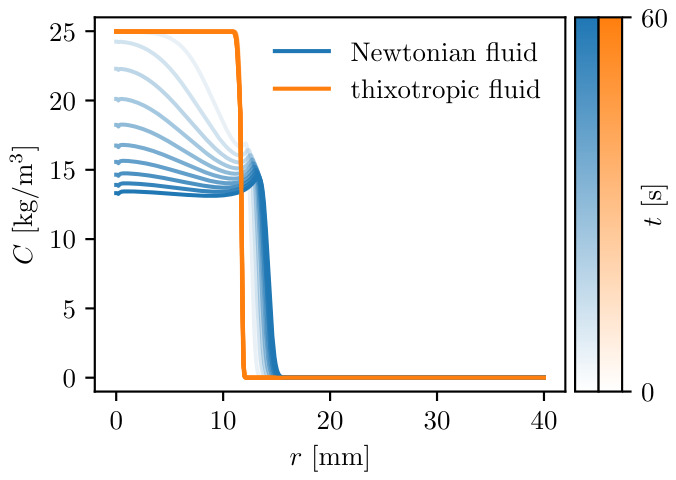
Concentration evolution in the Newtonian and thixotropic medication on the radius from the injection point in the first minute after the injection; the first line corresponds to 6 s after injection, the second to 12 s, etc.

The combined effects of reduced diffusion and reduced advection are clearly reflected in the concentration profiles shown in Figure 3. In the Newtonian case, the formulation spreads progressively through the tissue, resulting in a broadened concentration distribution and a decline in concentration near the injection site. In contrast, the thixotropic formulation preserves a sharper concentration profile and retains a larger fraction of the active substance within the initially treated region. These results highlight the critical importance of the first minute following injection, during which rapid viscosity recovery establishes the transport regime governing subsequent drug distribution.

Although approaches tailored for high concentration gradients (e.g., [16]) may appear more suitable, diffusion plays a comparatively minor role under the present conditions. Owing to the elevated pressure gradients generated by injection, transport is predominantly advection-driven, rendering diffusive contributions negligible in the analyzed regime.

### 3.2. First Hour and First Ten Hours

**Figure 4:**
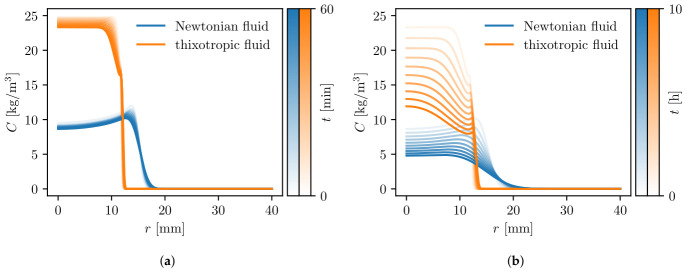
(**a**) Concentration evolution in the Newtonian and thixotropic medication on the radius from the injection point in the first hour after the injection; the first line corresponds to 6 min after injection, the second to 12 min, etc. (**b**) Concentration evolution in the Newtonian and thixotropic medication on the radius from the injection point in the first ten hours after the injection; the first line corresponds to 1 h after injection, the second to 2 h, etc.

The long-term effects of thixotropic recovery are illustrated by the concentration profiles in Figure 4. Figure 4a depicts the concentration evolution during the first hour after injection. Although both formulations exhibit gradual concentration decay due to biological elimination, the Newtonian formulation continues to spread within the tissue owing to its higher mobility. Consequently, the concentration peak diminishes more rapidly, while the affected region expands progressively.

The behavior of the thixotropic formulation differs markedly. Because of its elevated viscosity, advective transport is rapidly attenuated following injection. As a result, the concentration distribution remains localized near the injection site and the active substance is confined within a smaller tissue volume. This limited spatial spread implies that a greater fraction of the administered drug is retained within the target region, potentially enhancing therapeutic effectiveness while reducing exposure of surrounding healthy tissue.

The differences become even more pronounced over the first ten hours, as shown in Figure 4b. During this period, biological elimination becomes increasingly important and contributes to the overall reduction in concentration. Nevertheless, the thixotropic formulation continues to exhibit a more localized distribution than the Newtonian formulation. The results therefore suggest that thixotropic recovery acts as a passive retention mechanism that prolongs local residence time without requiring additional delivery devices or complex encapsulation strategies.

From a clinical perspective, this behavior is advantageous, as the primary objective of local chemotherapy is not only drug delivery but also the sustained maintenance of therapeutically effective concentrations within the tumor region. The model predictions indicate that thixotropic formulations support this objective by limiting drug dispersion while enhancing local retention, thereby potentially improving treatment efficacy and reducing off-target exposure.

### 3.3. Sensitivity Analysis

A sensitivity analysis was conducted to assess the influence of key tissue and formulation parameters on the spatial distribution of the active substance during the first hour after injection. Each parameter was varied around its baseline value while all others were held constant. The selected parameter values are summarized in Table 2, where the fourth row lists the baseline values used in the standard simulations and reported in Table 1.

It is important to note that, aside from 1.0 Pa·s and 50.0 Pa·s, the values for $\mu_{0}$ correspond to the available viscosities of Methocel Type K [19]. Furthermore, it should be emphasized that the equilibrium viscosity $\mu_{0}$ is not a fixed material constant but a formulation variable, tunable over the explored range through the choice of HPMC grade and polymer concentration. The optimal $\mu_{0}$ therefore depends on the clinical objective, namely the agent employed and the desired residence time, rather than being prescribed a priori. In this sense, the model itself functions as a design tool, which from a given target tumor radius and persistence, predicts the equilibrium viscosity and hence the formulation required to achieve it.

**Figure 5:**
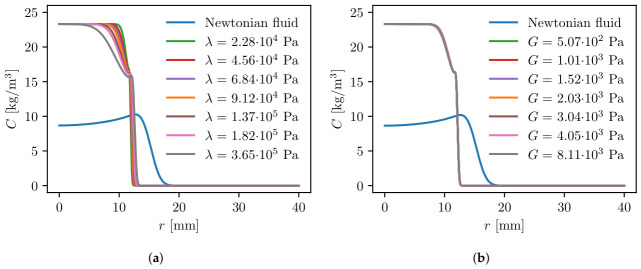
(**a**) Influence of the drained Lamé parameter λ on the drug concentration. (**b**) Influence of the shear modulus *G* on the drug concentration.

The tissue mechanical parameters (the drained Lamé parameter λ, the shear modulus *G* and the permeability *k*) act on the pressure evolution through the Biot poroelastic formulation and Darcy’s law. Across the investigated ranges, however, the volumetric and shear stiffness produce only minor variations in the predicted concentration fields, as shown in Figure 5, indicating that tissue stiffness is not a controlling factor for drug transport under the considered conditions.

**Figure 6:**
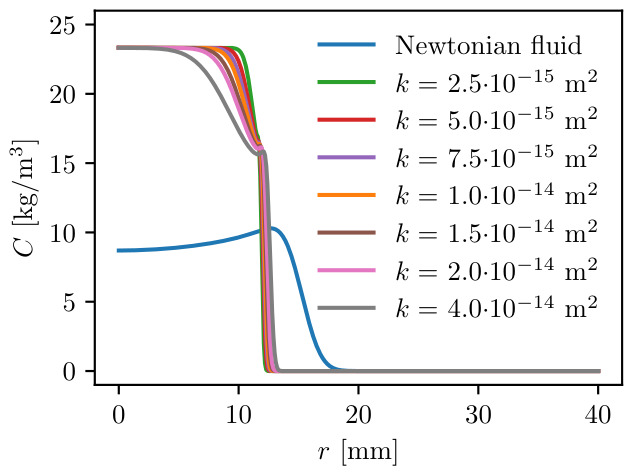
Influence of permeability *k* on the drug concentration.

Figure 6 reveals that the permeability *k* behaves in the same way, exerting an influence comparable to that of the Lamé parameter and producing only marginal changes in the concentration field. None of the tissue mechanical parameters is therefore a decisive factor for drug localization.

**Figure 7:**
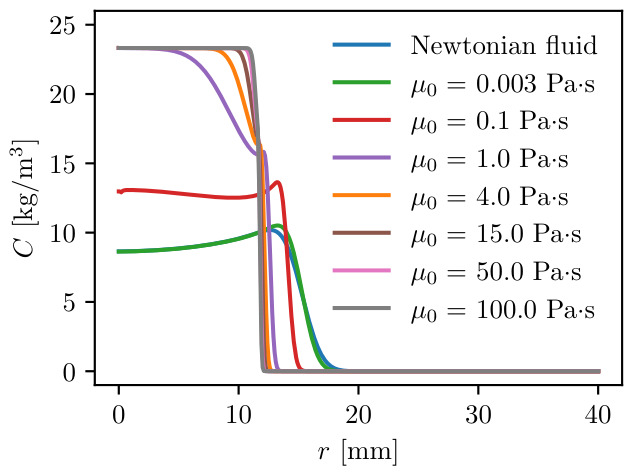
Influence of the equilibrium viscosity $\mu_{0}$ on the drug concentration.

In contrast, the thixotropic formulation parameters govern the viscosity evolution and therefore affect both diffusion and pressure-driven transport. The equilibrium viscosity $\mu_{0}$ is by far the most influential: increasing $\mu_{0}$ substantially limits the concentration drop caused by the initial pressure evolution, as shown in Figure 7, making it the key design parameter for thixotropic formulations in local chemotherapy.

**Figure 8:**
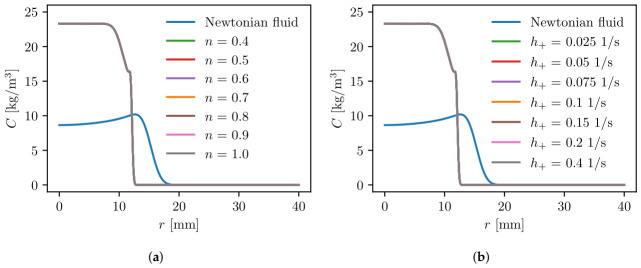
(**a**) Influence of the exponent *n* on the drug concentration. (**b**) Influence of the structure build-up parameter $h_{+}$ on the drug concentration.

Figure 8 exposes that the recovery-related parameters *n* and $h_{+}$ play only secondary roles: the exponent *n*, which sets the dependence of viscosity on structural recovery, has a comparatively weak effect that justifies the approximate value adopted in the model, while the build-up parameter $h_{+}$ mainly influences early-time transport, since most structural rebuilding occurs within the first minute after injection. Consequently, the equilibrium viscosity is more decisive than the precise recovery rate over the investigated timescales.

**Figure 9:**
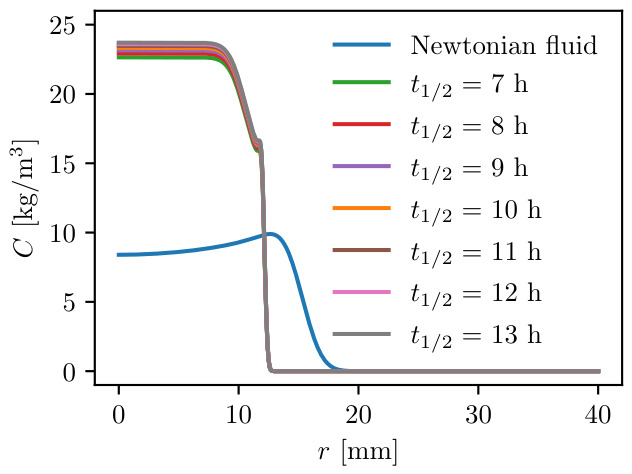
Influence of the biological half-life $t_{1/2}$ on the drug concentration.

Finally, the biological half-life $t_{1/2}$ differs in nature from the parameters above: rather than affecting fluid transport, it controls the rate at which the active substance is cleared from the tissue. Increasing $t_{1/2}$ therefore raises the concentration throughout the domain at later times, whereas shorter half-lives accelerate its decay, but in both cases the spatial distribution remains essentially unchanged, as shown in Figure 9. Biological elimination thus governs the magnitude of the concentration field, not the transport mechanisms shaping its spatial structure.

From a therapeutic standpoint, the two effects are complementary: the half-life sets the duration of pharmacological activity, while the thixotropic behavior governs spatial localization, so that their joint optimization is a promising route to more effective local chemotherapy. In summary, the sensitivity analysis identifies the equilibrium viscosity $\mu_{0}$ as the dominant parameter controlling drug localization, while the tissue mechanical properties induce only minor variations and the biological half-life acts solely on the overall concentration magnitude.

Altogether, the results trace a single causal chain; post-injection viscosity recovery suppresses pressure-driven advection and diffusion, which in turn confines the drug to a smaller radius and prolongs local retention. This interplay between viscosity recovery, transport suppression and tissue localization links the mechanical response, spreading and overall effectiveness of the treatment.

### 3.4. Quantitative Effectiveness Analysis

**Figure 10:**
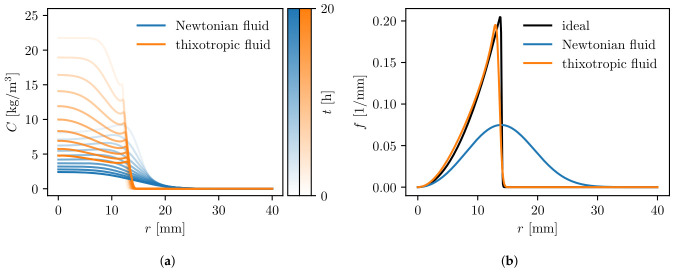
(**a**) Concentration evolution in the Newtonian and thixotropic medication on the radius from the injection throughout the simulation period of $2\cdot t_{1/2}$. (**b**) Auxiliary probability density functions at the end of the $2\cdot t_{1/2}$ time window taking into consideration a tumor radius of R=14 mm.

To enable a quantitative assessment of treatment effectiveness, additional constraints must be introduced. First, the tumor radius *R* is defined as the target treatment extent. In principle, the model would permit an unrealistically optimal scenario in which a precisely selected injected volume is combined with an arbitrarily large equilibrium viscosity $\mu_{0}$ to exactly match this radius. To avoid such non-physical solutions, the injected volume *V* is imposed as a fixed constraint. Furthermore, the analysis is restricted to a finite observation window of $2\cdot t_{1/2}$ following injection. The concentration evolution within this period, which captures the clinically relevant phase prior to substantial biological elimination, is shown in Figure 10a.

For the quantitative assessment of treatment effectiveness, both under- and over-spreading of the drug must be penalized. However, from a clinical standpoint, over-spreading, while potentially affecting healthy tissue, is preferable to under-spreading, which may leave malignant cells untreated. Accordingly, these two effects should be weighted asymmetrically within the evaluation metric. Furthermore, the absolute magnitude of the concentration profiles is of secondary importance, as the primary objective is to assess spatial distribution rather than total drug quantity. Owing to mass conservation, profiles that reach the same spatial extent cannot differ significantly in total content. To address these requirements, a measure from information theory is adopted. The Kullback–Leibler (KL) divergence, or relative entropy, quantifies the discrepancy between an approximating distribution *X* and a reference distribution *Y*. Its directional nature inherently introduces asymmetry, allowing under-spreading to be penalized more strongly than over-spreading. In addition, because it operates on normalized distributions, it emphasizes spatial differences while suppressing the influence of absolute magnitude. For continuous distributions *X* and *Y*, the KL divergence is defined as

(42) $$D_{\mathrm{KL}}\left(X\|Y\right)=\int_{-\infty }^{\infty }x\left(s\right)\;\ln\frac{x(s)}{y(s)}\;ds,$$

where x(s) and y(s) denote the probability density functions of *X* and *Y*. Keeping in mind the directionality of the KL divergence, we can write

(43) $$x\left(r\right)=\left\{\begin{array}{cc} \frac{3}{R^{3}}\;r^{2}, & r\leq R,\\ 0, & r>R \end{array}\right.$$

and

(44) $$y\left(r,t\right)=\frac{C\left(r,t\right)\;r^{2}}{\int_{0}^{\infty }C\left(r,t\right)\;r^{2}\;dr}.$$

To illustrate this approach, the corresponding auxiliary probability density functions are evaluated at the end of the $2\cdot t_{1/2}$ observation window, assuming a tumor radius of R=14 mm. The resulting distributions are shown in Figure 10b, where *f* is used as a placeholder for both x(r) (ideal reference distribution) and y(r) (distributions obtained for the Newtonian and thixotropic formulations).

Nothing restricts the inclusion of temporal dependence in the evaluation metric, therefore, the effectiveness of the therapy is defined as a time-dependent functional, J(t), given by

(45) $$J\left(t\right)=\int_{0}^{\infty }x\left(r\right)\ln\frac{x(r)}{y(r,t)}dr.$$

The effectiveness is evaluated over the previously defined time window of $2\cdot t_{1/2}$. First, the baseline concentration evolutions are analyzed for varying tumor radii *R*, followed by a parametric study in which the equilibrium viscosity $\mu_{0}$ is varied for a fixed *R*. It should be noted that, due to the definition of J(t) and the reference distribution x(r), careful numerical treatment of singularities (e.g., ln0 and division by zero) is required.

**Figure 11:**
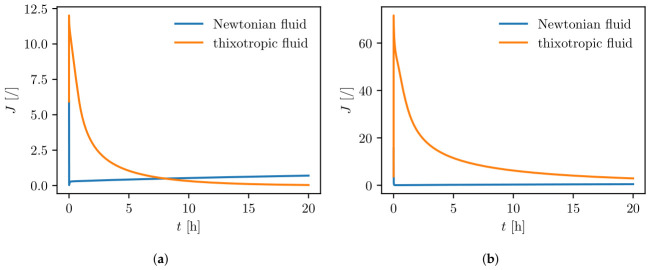
(**a**) Effectiveness of the Newtonian and thixotropic medication for the case of a tumor radius of R=14 mm throughout the simulation period of $2\cdot t_{1/2}$. (**b**) Effectiveness of the Newtonian and thixotropic medication for the case of a tumor radius of R=16 mm throughout the simulation period of $2\cdot t_{1/2}$.

The results of the effectiveness analysis for the representative case of R=14 mm are presented in Figure 11a.

It is evident that, for cases in which the active substance remains present in the tissue for more than approximately 8 h (a condition satisfied in the present simulations), the thixotropic formulation consistently outperforms the Newtonian counterpart. This is attributable to its enhanced retention and reduced spatial dispersion. Furthermore, the effectiveness curves exhibit the expected asymmetric behavior, penalizing under-spreading more strongly than over-spreading, thereby producing a well-defined minimum. The time of this minimum corresponds to the optimal duration of the therapy. The results of the effectiveness analysis for the case of a tumor radius of R=16 mm are presented in Figure 11b.

**Figure 12:**
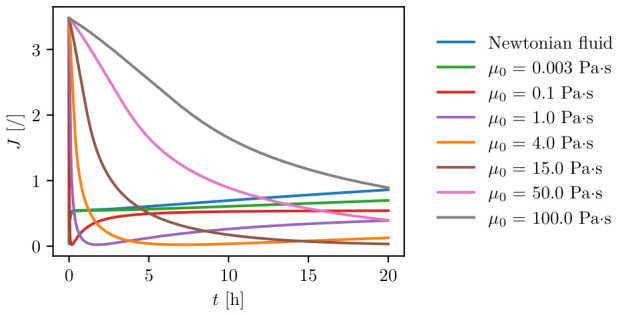
Effectiveness of the Newtonian and thixotropic medications with different equilibrium viscosities $\mu_{0}$ for the case of a tumor radius of R=13 mm throughout the simulation period of $2\cdot t_{1/2}$.

In this case, the results indicate that, within the prescribed therapeutic time window, the Newtonian formulation remains more effective. This outcome reflects the balance between drug retention and spatial coverage, where increased spreading enhances target coverage despite reduced localization. Finally, to further investigate the role of rheological properties, the tumor radius is fixed at R=13 mm and the effectiveness of formulations with varying equilibrium viscosities $\mu_{0}$ is evaluated. The corresponding results are presented in Figure 12.

In this case, the results indicate that toward the end of the considered time window, corresponding closely to the effective persistence of the active substance within the tissue, a formulation with an equilibrium viscosity of $\mu_{0}=15.0$ Pa·s provides superior performance compared to the previously examined thixotropic formulation with $\mu_{0}=4.0$ Pa·s. This finding highlights the strong influence of equilibrium viscosity on long-term drug retention and spatial confinement.

### 3.5. Model Limitations

Although the proposed formulation provides valuable insight into the transport behavior of thixotropy-enhanced chemotherapy delivery, several simplifying assumptions were introduced to maintain mathematical tractability. These can be grouped into four categories: geometric simplification, tissue medium simplification, rheological and pharmacokinetic simplification and the absence of experimental validation.

The model assumes spherical symmetry around the injection site. The spherical reduction is adopted deliberately as a physically transparent first iteration—it retains the dominant coupling between rheological recovery and transport while keeping the numerics manageable, allowing the underlying physics to be understood before additional geometric and biological complexity is introduced. Tumor heterogeneity, irregular vascularization and anisotropic permeability would all alter local distribution and would substantially complicate the numerical treatment, potentially obscuring the very mechanisms isolated here.

The tissue was modeled as a homogeneous poroelastic medium with constant permeability, porosity, tortuosity and constrictivity. In reality, biological tissues are heterogeneous and may contain vascular and lymphatic networks, as well as structural interfaces, all of which can significantly alter local transport conditions. Tumor tissue, in particular, often exhibits pronounced spatial variability in both mechanical properties and hydraulic conductivity. The magnitude of tissue deformation near the injection site introduces a further limitation since the model predicts an initial volumetric strain of approximately 15 %, which approaches the upper validity range of the small-deformation assumption inherent to linear Biot poroelasticity. While this linear framework is sufficient for capturing the dominant transport mechanisms and enabling comparative analysis of different formulations, a finite-strain poroelastic formulation would be required for strictly accurate quantitative predictions in the near-field region.

The constitutive description of thixotropy was based on an idealized structural parameter model. While this formulation captures the essential feature of post-injection viscosity recovery, the rheological behavior of real pharmaceutical systems may involve additional phenomena, including viscoelasticity, shear-thinning, yield stress and multi-stage structural rebuilding, so experimental rheological characterization would be required for accurate quantitative predictions of specific formulations. The active substance was likewise treated as a passive solute, so that key biological processes (cellular uptake, receptor binding, intracellular accumulation and interactions with the extracellular matrix) are not accounted for and drug elimination is represented by a simplified first-order decay model based on the half-life, whereas actual pharmacokinetics may depend on local perfusion, metabolism and tissue-specific clearance mechanisms. Finally, vascular and lymphatic transport pathways are neglected—both mechanisms can play a significant role in highly vascularized tissues and may substantially influence long-term drug distribution and their incorporation would require coupling the present framework with additional transport equations describing fluid and solute exchange between the tissue and the circulatory system.

As the present work is focused on theoretical model development and numerical analysis, experimental validation has not been performed and a direct quantitative comparison with published intratumoral injection data is presently unfeasible as time-dependent thixotropic structural recovery has not been incorporated in prior intratumoral transport models and the rapid post-injection dispersion of unthickened drug solutions reported in the experimental literature is qualitatively consistent with the Newtonian limit of the present framework. A systematic, quantitative validation is therefore envisaged as a staged program: controlled injections into tissue phantoms and artificial gel matrices to verify the predicted spreading and viscosity-recovery dynamics, followed by ex vivo and in vitro studies and ultimately in vivo measurement. This program would proceed in parallel with the geometric and biological generalization outlined above, which will also reveal whether more elaborate models capture phenomena not represented in the present formulation.

Despite these limitations, the model successfully captures the key physical mechanisms governing the interaction between thixotropic recovery, porous medium transport and drug distribution. The proposed framework thus provides a useful foundation for future experimental studies and for the rational design and optimization of localized chemotherapy formulations.

## 4. Conclusions

A mathematical framework was developed to investigate the transport and retention of locally injected chemotherapeutic agents delivered using thixotropic polymer carriers. By coupling a structural recovery model for HPMC-based formulations with Darcy flow, Biot poroelasticity and advection–diffusion transport, the formulation captures the interplay between rheological evolution and drug distribution within porous tumor tissue.

The simulations demonstrate that rapid post-injection viscosity recovery markedly suppresses pressure-driven transport and enhances local drug retention. Among the investigated parameters, the equilibrium viscosity emerges as the dominant factor governing concentration localization, while tissue mechanical properties and recovery kinetics exert comparatively minor influence over the considered timescales.

Importantly, the results indicate that increased retention is not universally advantageous. The effectiveness analysis reveals a tumor-size-dependent trade-off between localization and spatial coverage. For intermediate tumor sizes, thixotropic recovery improves confinement within the target region, whereas for larger tumors, excessive viscosity can hinder transport and result in insufficient coverage. These findings indicate that optimal carrier rheology must be tailored to tumor dimensions.

The proposed framework thus provides a physically grounded basis for analyzing the relationship between carrier rheology and local drug distribution. Future work should focus on experimental validation, extension to finite-strain poromechanics, and incorporation of vascular and lymphatic transport pathways, and aim for a geometric and biological generalization. Such developments will enable even more accurate prediction of clinically relevant scenarios and support the rational design of hydrogel-assisted local chemotherapy strategies.

## Figures and Tables

**Table 1:** Parameter values for the simulations.

Parameter	Value	Unit	Reference
$\mu_{N}$	$1.5\times 10^{-3}$	Pa·s	[20]
$\mu_{0}$	4.0	Pa·s	[19]
*n*	0.7	/	approximate value
$h_{+}$	0.10	1/s	approximate value
*k*	$1.0\times 10^{-14}$	m^2^	[25]
λ	$9.12\times 10^{4}$	Pa	[26]
*G*	$2.03\times 10^{3}$	Pa	[26]
*V*	$1.0\times 10^{-6}$	m^3^	[27]
φ	0.15	/	[28]
δ	0.85	/	[29]
τ	1.41	/	[28,29]
*T*	310.15	K	body temperature
ρ	1500.0	kg/m^3^	[30]
*M*	0.45444	kg/mol	[23]
$t_{1/2}$	36,000.0	s	[31]
$C_{0}$	25.0	kg/m^3^	[31]

**Table 2:** Parameter values for the sensitivity analysis.

λ [Pa]	*G* [Pa]	*k* [m^2^]	$\mu_{0}$ [Pa·s]	*n* [/]	$h_{+}$ [1/s]	$t_{1/2}$ [s]
$2.28\times 10^{4}$	$5.07\times 10^{2}$	$2.5\times 10^{-15}$	0.003	0.4	0.025	25,200
$4.56\times 10^{4}$	$1.01\times 10^{3}$	$5.0\times 10^{-15}$	0.1	0.5	0.050	28,800
$6.84\times 10^{4}$	$1.52\times 10^{3}$	$7.5\times 10^{-15}$	1.0	0.6	0.075	32,400
$9.12\times 10^{4}$	$2.03\times 10^{3}$	$1.0\times 10^{-14}$	4.0	0.7	0.10	36,000
$1.37\times 10^{5}$	$3.04\times 10^{3}$	$1.5\times 10^{-14}$	15.0	0.8	0.15	39,600
$1.82\times 10^{5}$	$4.05\times 10^{3}$	$2.0\times 10^{-14}$	50.0	0.9	0.20	43,200
$3.65\times 10^{5}$	$8.11\times 10^{3}$	$4.0\times 10^{-14}$	100.0	1.0	0.40	46,800

**Figure A1:**
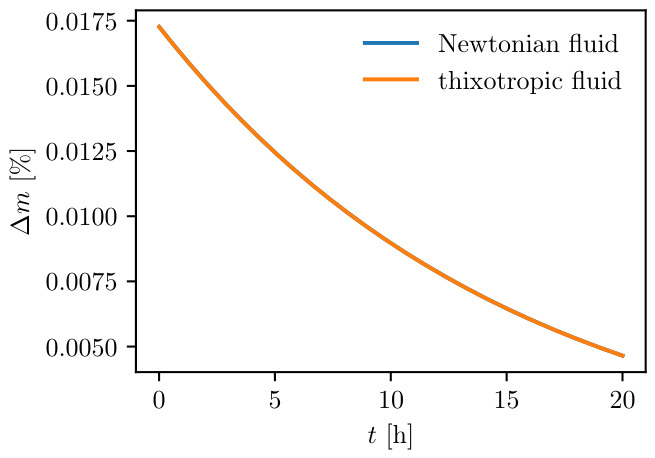
Relative mass conservation error Δm(t) throughout the simulation period of $2\cdot t_{1/2}$.

**Figure A2:**
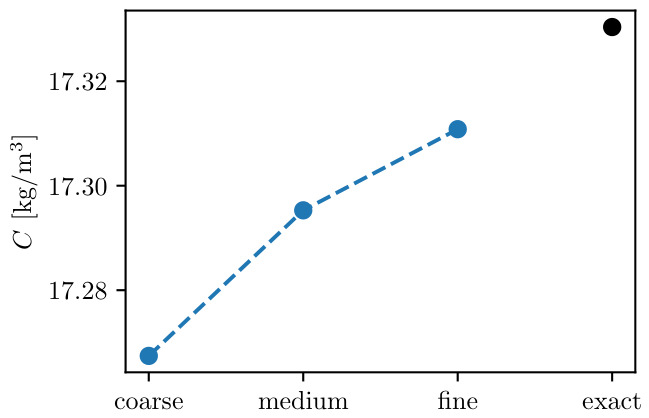
Results of the Richardson extrapolation.

## Data Availability

The original contributions presented in this study are included in the article. Further inquiries can be directed to the corresponding author.

## Abbreviations

The following abbreviations are used in this manuscript:

DNA	Deoxyribonucleic acid
HPMC	Hydroxypropyl methylcellulose
MTX	Methotrexate

## Appendix A

Here, we present the relative mass conservation error throughout the simulation period. Because of the existing decay, the error Δm(t) is calculated as

(A1) $$\Delta m\left(t\right)=\frac{m\left(t\right)-m^{\prime }\left(t\right)}{m_{0}},$$

where m(t) and $m^{\prime }\left(t\right)$ are the actual and the expected total mass, while $m_{0}=25.0$ mg is the initial total mass. The actual mass is tracked through the simulations and the expected mass is calculated based on the prescribed decay due to the biological half-life of the medication:

(A2) $$m^{\prime }\left(t\right)=m_{0}\;e^{S_{0}\;t}.$$

The relative mass conservation error through time is shown in Figure A1.

## Appendix B

Here, we present the results of the Richardson extrapolation. Since the the first few seconds of the analysis represent the most dynamic behavior, especially in the case of the Newtonian medication, it makes sense to choose its concentration at r=10 mm and t=10 s as the reference value for the Richardson extrapolation. The analysis was performed with a spatial discretization of $2.0\times 10^{-4}$ m, $1.0\times 10^{-4}$ m and $0.5\times 10^{-4}$ m and its results are shown in Figure A2.

Finally, all the values are presented in Table A1.

**Table A1:** Values of the Richardson extrapolation.

Mesh	*C* [kg/m^3^]	RDE [%]
coarse	17.27	−0.36
medium	17.30	−0.20
fine	17.31	−0.11

We can safely assume that our spatial step (corresponding to that of the medium grid) was small enough to correctly capture even the most dynamic behavior.
